# LONGITUDINAL REPORTS OF NEIGHBORHOOD ENVIRONMENT ON COGNITIVE PERFORMANCE AMONG BLACK ADULTS

**DOI:** 10.1093/geroni/igad104.2678

**Published:** 2023-12-21

**Authors:** Alexa Allan, Alyssa Gamaldo, Roland J Thorpe, Jennifer D Roberts, Michele K Evans, Alan B Zonderman

**Affiliations:** The Pennsylvania State University, University Park, Pennsylvania, United States; Clemson University, Clemson, South Carolina, United States; Johns Hopkins University, Baltimore, Maryland, United States; University of Maryland, College Park, Maryland, United States; National Institute on Aging, National Institutes of Health, Baltimore, Maryland, United States; National Institute on Aging, National Institutes of Health, Baltimore, Maryland, United States

## Abstract

Limited research has explored the association between neighborhood environments and cognitive functioning using longitudinal reports of neighborhood quality. The current study examined the longitudinal association between perceived neighborhood characteristics and computerized cognitive performance among Black adults. This study included 584 community-dwelling socioeconomically diverse Black adults (Mage = 56.27, SDage = 8.94; 58% female) from waves 3 & 4 of the Healthy Aging in Neighborhood of Diversity across the Life Span (HANDLS) study. Neighborhood quality was assessed using three scales about built environment disorder (e.g., litter), social cohesion (e.g., close-knit neighborhood), and social control (e.g., neighbors act if fight in front of house). Cognitive functioning was assessed using the computerized Joggle battery. Multivariable linear regression analyses were conducted with each cognitive measure as the outcome measure. Models were adjusted for age, sex, education, reading literacy, poverty status, and depressive symptoms. In the adjusted models, living in a neighborhood with increasing built environment disorder was associated with slower performance (p <.05) on an attention measure. Increasing social cohesion was associated with faster performance (p <.05) on memory, attention, and visual orientation measures. Similarly, increasing social control was associated with faster performance (p <.05) on attention, executive function/reasoning, visual orientation, and speed measures. Additionally, increasing social control was associated with greater number of correct responses (p <.05) on an attention and executive function/reasoning measure. Significant interactions were observed between changes in neighborhood quality and covariates. These findings amplify the importance of understanding how changes in neighborhood quality impact cognitive performance among Black adults.

